# AI-driven personalized nutrition: RAG-based digital health solution for obesity and type 2 diabetes

**DOI:** 10.1371/journal.pdig.0000758

**Published:** 2025-05-06

**Authors:** Anand K. Gavai, Jos van Hillegersberg

**Affiliations:** 1 Industrial Engineering & Business Information Systems, University of Twente, Enschede, The Netherlands; 2 Jheronimus Academy of Data Science, ‘s-Hertogenbosch, The Netherlands; 3 Department of Mathematics and Computer Science, Eindhoven University of Technology, Eindhoven, The Netherlands; Shahid Beheshti University of Medical Sciences School of Dentistry, IRAN, ISLAMIC REPUBLIC OF

## Abstract

Effective management of obesity and type 2 diabetes is a major global public health challenge that requires evidence-based, scalable personalized nutrition solutions. Here, we present an artificial intelligence (AI) driven dietary recommendation system that generates personalized smoothie recipes while prioritizing health outcomes and environmental sustainability. A key feature of the system is the “virtual nutritionist”, an iterative validation framework that dynamically refines recipes to meet predefined nutritional and sustainability criteria. The system integrates dietary guidelines from the National Institute for Public Health and the Environment (RIVM), EUFIC, USDA FoodData Central, and the American Diabetes Association with retrieval-augmented generation (RAG) to deliver evidence-based recommendations. By aligning with the United Nations Sustainable Development Goals (SDGs), the system promotes plant-based, seasonal, and locally sourced ingredients to reduce environmental impact. We leverage explainable AI (XAI) to enhance user engagement through clear explanations of ingredient benefits and interactive features, improving comprehension across varying health literacy levels. Using zero-shot and few-shot learning techniques, the system adapts to user inputs while maintaining privacy through local deployment of the LLaMA3 model. In evaluating 1,000 recipes, the system achieved 80.1% adherence to health guidelines meeting targets for calories, fiber, and fats and 92% compliance with sustainability criteria, emphasizing seasonal and locally sourced ingredients. A prototype web application enables real-time, personalized recommendations, bridging the gap between AI-driven insights and clinical dietary management. This research underscores the potential of AI-driven precision nutrition to revolutionize chronic disease management by improving dietary adherence, enhancing health literacy, and offering a scalable, adaptable solution for clinical workflows, telehealth platforms, and public health initiatives, with the potential to significantly alleviate the global healthcare burden.

## Summary

Managing obesity and type 2 diabetes can be overwhelming, especially when nutrition advice feels complex or generic. In this study, we introduce a personalized and easy-to-use digital tool that helps people take control of their health—one smoothie at a time. Using artificial intelligence, our system creates tailored smoothie recipes based on each user’s age, body size, and preferences. These recipes aren’t just healthy—they’re designed to meet established medical guidelines while also being sustainable, using seasonal and locally sourced ingredients. We’ve built in a “virtual nutritionist” that checks and refines each recipe to ensure it aligns with nutrition and environmental goals. Unlike many AI tools, ours runs locally, keeping personal data private and secure. In testing, 80% of the recipes met strict nutritional targets, and 92% used sustainable ingredients. A web app lets users receive their personalized recipes in real time, with clear explanations for every choice made. Our work shows how AI can translate expert advice into everyday actions, supporting healthier lifestyles and empowering people to make better food choices—no matter their level of health knowledge.

## Introduction

Obesity and type 2 diabetes are significant global health challenges, affecting millions and placing immense strain on healthcare systems worldwide. In the Netherlands, over 50% of adults are classified as overweight, and approximately 1.2 million individuals live with type 2 diabetes [1,2]. These alarming statistics highlight the urgent need for scalable nutritional interventions that not only regulate blood sugar levels and support weight management but can also be integrated into clinical workflows to improve patient outcomes and reduce healthcare costs.

Dietary management is fundamental in controlling obesity and type 2 diabetes. Despite general dietary guidelines, many individuals struggle to adhere to these recommendations due to a lack of personalization. Standard dietary advice often fails to account for individual health status, lifestyle, and dietary preferences. Additionally, people with low health literacy may find it challenging to interpret and implement complex dietary guidelines, leading to poor adherence and less effective management of chronic conditions. This highlights a critical gap in existing digital health tools, which often fail to provide real-time, personalized dietary recommendations that are both clinically accurate and user-friendly. By integrating AI-driven personalization with evidence-based data, this study bridges this gap, offering a solution that is scalable and adaptable to diverse healthcare settings. Therefore, accessible and understandable personalized nutritional advice is crucial for individuals with varying levels of health literacy.

Advances in artificial intelligence (AI), especially large language models like GPT-4 [3,4] and LLaMA [5] provide new opportunities for personalized dietary recommendations. LLMs excel at transforming complex health information into simplified, human-friendly language, enhancing health literacy by presenting facts in an easily comprehensible format. By translating expert dietary guidelines into understandable terms, LLMs bridge the gap between medical knowledge and individual comprehension, enabling more effective dietary management. However, the challenge remains that LLMs often rely on unverified internet sources, posing risks in healthcare applications [6]. To mitigate this, it is essential to integrate these models with reliable, evidence-based data sources.

The Retrieval-Augmented Generation (RAG) framework [7] addresses this issue by combining the generative power of LLMs with a retrieval mechanism that accesses trusted databases. This approach ensures the generation of personalized dietary advice that is scientifically sound, contextually relevant, and tailored to individual needs. Moreover, the system can dynamically adjust the complexity of the recommendations based on user input, making the information accessible to people with diverse health literacy levels. By retrieving and integrating data from validated sources, the system provides accurate, personalized, and easy-to-implement dietary recommendations.

This study presents an AI-driven dietary recommendation system built on the RAG framework, specifically designed to generate personalized smoothie recipes for individuals with obesity and type 2 diabetes. Smoothies are an ideal medium for personalized nutrition due to their flexibility and adaptability [8,9]. They can incorporate a wide variety of nutrient-rich ingredients—such as fruits, vegetables, seeds, and nuts—allowing for precise customization to achieve specific health goals, such as reducing the glycemic index (GI), increasing fiber intake, and maintaining a balanced macronutrient profile. This versatility makes smoothies suitable for individuals with chronic conditions, offering a convenient and appealing way to follow tailored dietary plans. Additionally, the simplicity of smoothie preparation caters to those who may lack cooking skills, time, or sufficient health literacy, further promoting adherence.

Considering the environmental impact of global food systems, sustainability is an increasingly important aspect of food policy. Integrating sustainability principles into personalized nutrition ensures that dietary recommendations promote health while minimizing environmental footprints. This system aligns with the Dutch dietary guidelines from the National Institute for Public Health and the Environment (RIVM) [10] and incorporates sustainability principles [11] to ensure that the personalized smoothie recipes are both nutritionally appropriate and environmentally sustainable. By promoting plant-based ingredients, seasonal availability, and locally sourced food, the system supports sustainable food practices that contribute to individual health and environmental goals utilizing data from eufic that provides list of sustainable and seasonal fruits and vegetables for all european countries (www.eufic.org).

The development of this system aligns with multiple United Nations Sustainable Development Goals (SDGs). Beyond its primary focus on SDG 3 (Good Health and Well-being) through diabetes and obesity management, the system contributes to SDG 2 (Zero Hunger) by promoting nutritious food choices, SDG 12 (Responsible Consumption and Production) through its emphasis on sustainable ingredients, and SDG 13 (Climate Action) via its low-carbon footprint approach to dietary recommendations. Furthermore, the system embraces circular economy principles in food systems by prioritizing whole ingredient usage, minimizing food waste through precise portioning, and promoting local food networks [12].

To enable practical use, a prototype web application was developed to allow users to create personalized smoothie recipes tailored to their health profiles. The application adjusts the information complexity based on user preferences, ensuring alignment with the user’s health literacy. The full source code for this application is publicly available on Zenodo (https://doi.org/10.5281/zenodo.13898086).

**Fig 1:**
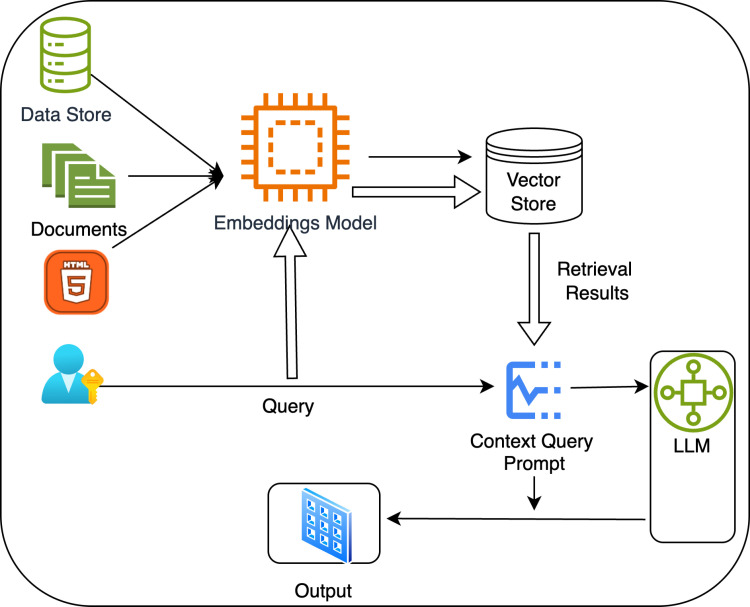
System architecture of the retrieval-augmented generation model for personalized smoothie recipes.

By integrating personalized health recommendations, sustainability considerations, and support for varying health literacy levels, this research advances nutritional interventions for managing obesity and type 2 diabetes while supporting broader environmental sustainability goals. Fig 1 illustrates the system architecture, showing how AI and retrieval mechanisms work together to provide user-specific dietary recommendations based on health literacy and evidence-based data. This system not only addresses individual dietary needs but also offers a scalable solution to reduce the burden of obesity and type 2 diabetes on global healthcare systems, aligning with public health priorities and the Sustainable Development Goals. While recent studies have explored personalized nutrition using large language models (LLMs) and optimization techniques, few have integrated sustainability considerations, explainable reasoning, and real-time validation. This study builds upon emerging work in AI-enhanced food recommendation systems, offering a uniquely comprehensive framework that combines evidence-grounded retrieval, personalization, and local privacy-preserving deployment. To contextualize our contributions, we discuss relevant work in personalized nutrition and AI in the related work section below.

The following sections detail the system’s development, validation, and impact on chronic disease management. Our results demonstrate that the system consistently generates recipes aligned with dietary guidelines for obesity and type 2 diabetes while promoting sustainable dietary practices and enhancing health literacy.

### Related work

Recent advances in AI-driven dietary recommendation systems highlight the importance of personalization, health optimization, and explainability. MOPI-HFRS [13] introduced a multi-objective framework for food recommendation, using LLMs to interpret user preferences and health constraints. However, it does not incorporate sustainability metrics or real-time nutrient retrieval from external sources.

NGQA [14] proposed a benchmark for nutritional question answering using graph structures. While it enhances reasoning over dietary data, it is not optimized for real-time recommendations or integration with personalized input such as BMI or dietary preferences.

Diet-Odin [15] focused on interpretable dietary patterns in the context of opioid misuse. Although not nutrition-specific, its explainability architecture is methodologically relevant. In contrast, our system combines retrieval-augmented generation with a virtual validation layer (“virtual nutritionist”) to create health-compliant, sustainable, and personalized smoothie recipes.

To our knowledge, this is the first implementation of a locally-deployed LLM-based dietary system using real-time nutrient APIs, explainable AI, and sustainability validation, tailored for type 2 diabetes and obesity management.

## Materials and methods

Given the reliance on publicly available data, the system ensures compliance with ethical standards by processing all data locally, avoiding cloud-based storage to minimize privacy risks. Robust encryption protocols safeguard sensitive inputs like age, BMI, and dietary preferences. Additionally, the system adheres to GDPR principles, ensuring user consent and data protection through anonymization and secure handling. These measures align with ethical guidelines for digital health innovations and emphasize the system’s commitment to data privacy.

### Data sources

The personalized smoothie recipe creator utilizes curated datasets to ensure nutritional accuracy, sustainability, and relevance for individuals with obesity and type 2 diabetes:

- **Dutch Dietary Guidelines for Obesity and Type 2 Diabetes**: This dataset, from the Dutch National Institute for Public Health and the Environment (RIVM) (Advisory Report on Dutch Dietary Guidelines for type 2 diabetics, n.d.), focuses on optimal macronutrient distribution, glycemic index control, fiber intake, and dietary patterns for managing these health conditions. The availability of Dutch dietary guidelines in English enables seamless integration into the RAG system, ensuring accuracy without requiring translations.
- **Sustainable Diet Guidelines**: These guidelines, also from the RIVM (The environmental sustainability of Dutch diet, n.d.), promote a plant-based, seasonal, and low-impact diet. They are integrated into the system to align recipes with health and environmental sustainability goals.
- **EUFIC Seasonal and Local Produce Database**: The system incorporates data from the European Food Information Council (EUFIC), which provides a comprehensive list of fruits and vegetables that are locally grown and seasonally available across European countries. This ensures that the personalized smoothie recipes prioritize ingredients that are sustainable, locally sourced, and in season, reducing the environmental impact associated with long-distance food transport.
- **Curated Smoothie Recipes**: A collection of diabetes-friendly smoothie recipes, cross-referenced with the USDA FoodData Central database [16], ensures accuracy in terms of calories, carbohydrates, fiber, fats, and sodium. Additional details on Dutch dietary guidelines, curated smoothie recipes, and system prompts can be found in supporting information files S1–S4 Text, providing essential context for the data sources and system design.

### Assigning ingredients and retrieving nutritional data

To ensure that the system creates accurate nutritional profiles, a predefined ingredient list has been created that maps each ingredient to the corresponding entry in the USDA FoodData Central database. This mapping ensures, detailed nutritional information can be retrieved in real time. The nutritional information (e.g., calories, carbohydrates, fiber, protein, fat and sodium) for the selected ingredients is dynamically retrieved from the USDA database. If the data is not available or the retrieval process encounters a problem, default nutritional information is used to maintain continuity in recipe creation. This approach ensures that the system uses up-to-date and reliable nutritional information for each ingredient.

### Selection of seasonal ingredients

The system uses EUFIC data to prioritize seasonal fruits and vegetables, ensuring sustainability by reducing the environmental impact of food production. By selecting locally available, in-season ingredients, the system promotes eco-friendly and nutritionally balanced recipes.

### Contextual data retrieval for personalized recipes

To ensure that the generated recipes are contextualized, a database of embedded documents is used. These documents include nutritional guidelines, smoothie recipes and sustainability principles. Based on user input such as age, BMI and dietary preferences, relevant information is retrieved from this database. This contextual data helps personalize smoothie recipes to meet the specific health needs of individuals struggling with obesity and type 2 diabetes.

### Smoothie recipe generation

The smoothie recipe generation system personalizes recipes by combining user inputs—such as age, BMI, and dietary preferences—with seasonal ingredients. Dietary restrictions and taste preferences are incorporated by filtering ingredients to meet user-specific criteria while adhering to nutritional and sustainability guidelines.

The machine learning model, LLaMA3, generates the recipes, including ingredient lists, nutritional breakdowns, and preparation instructions. LLaMA3 was selected because it runs locally, ensuring data privacy and compliance with ethical standards by avoiding cloud-based server dependencies. This feature also supports seamless integration into clinical workflows, where data privacy and regulatory compliance are critical. Other models, like GPT-4, were considered but excluded due to higher computational costs and privacy concerns. LLaMA3 offers an optimal balance of accuracy, efficiency, and explainability. Additionally, alternative approaches such as rule-based engines or generative-only LLMs (e.g., GPT-4 without retrieval) were explored. Rule-based systems lack flexibility in adapting to individual health profiles, while standalone generative models risk hallucinating facts—a critical concern in healthcare. By combining a retrieval step grounded in authoritative sources (e.g., USDA, RIVM) with generation, the RAG approach ensures that all recommendations are both personalized and evidence-based, making it particularly suitable for health-sensitive applications.

To ensure accuracy, the system retrieves real-time nutrient data for the selected seasonal ingredients from the USDA FoodData Central database. The nutritional content is then aligned with dietary guidelines. Explainability is achieved by providing users with a detailed nutritional breakdown, insights into the health benefits of key ingredients, and information on their seasonal availability, enhancing both understanding and adherence to recommendations. The system uses explainable AI (XAI) to provide users with detailed insights into the nutritional and health benefits of each recipe. This approach ensures transparency, enhances user understanding, and builds trust by explaining how ingredient choices align with health goals and dietary guidelines.

**Fig 2:**
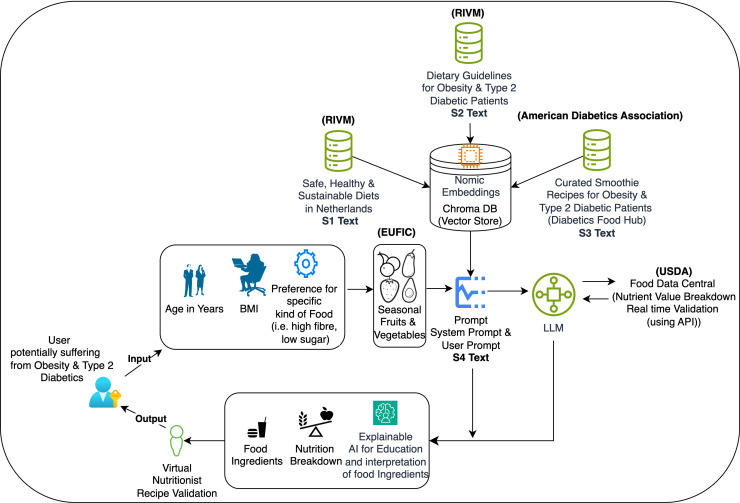
Illustration of the smoothie generation system’s workflow, showing how user inputs, seasonal ingredients, and validation processes integrate to create personalized and sustainable dietary recommendations. Supporting information used in this system includes sustainable dietary guidelines (S1 Text), Dutch diabetes guidelines (S2 Text), curated smoothie recipes (S3 Text), and prompt examples for recipe generation (S4 Text).

The full system architecture, which illustrates how the components (user input, contextual data retrieval, recipe creation and validation) work together, is shown in Fig 2.

### Zero-shot and few-shot learning

Zero-shot and few-shot learning [17] enable the system to generate personalized recipes with minimal training data. Zero-shot learning generalizes based on pre-trained knowledge, while few-shot learning uses small contextual examples, ensuring flexibility and adaptability to user inputs:

- **Zero-Shot Learning:** The model leverages its pre-trained knowledge to perform tasks it has never explicitly seen during training, generalizing based on minimal information provided at inference time. In the smoothie generation system, the LLM uses user inputs (age, BMI, dietary preferences) and context from ChromaDB (e.g., dietary guidelines) to generate personalized recipes without being trained on every scenario.
- **Few-Shot Learning:** This approach provides the model with a small number of examples or hints to fine-tune its task understanding. In the system, contextual documents from ChromaDB (such as sustainable diet guidelines and nutritional information) serve as minimal examples, guiding the model to generate more accurate responses.

By combining these techniques, the system creates flexible, personalized outputs, even in novel situations, enhancing adaptability and relevance.

### Validation framework

The validation framework ensures adherence to Dutch dietary guidelines and sustainability standards. The “virtual nutritionist” dynamically refines recipes during generation to guarantee 100% compliance with predefined criteria, ensuring reliability and adaptability. Separately, the validation model evaluates the large language model (LLM) independently of the virtual nutritionist. This enables objective assessment of how the LLM generates recipes without iterative refinement, highlighting areas for potential improvement. The framework also maintains transparency with auditable thresholds and logs, enabling integration into clinical workflows.These guidelines focus on glycemic control, weight management, and environmental sustainability.

While the virtual nutritionist iteratively refines recipes to meet predefined criteria, the validation framework serves as an independent assessment to ensure transparency, reliability, and adherence to guidelines. It quantifies success rates, identifies improvement areas, and ensures adaptability to real-world scenarios, complementing the virtual nutritionist’s automated checks while supporting user trust and regulatory compliance.

#### Nutritional validation.

Recipes are assessed against the following thresholds:

- **Calories**: ≤ 350 kcal per serving
- **Carbohydrates**: ≤ 50 g per serving
- **Fiber**: ≥ 8 g per serving
- **Sugar and Fat**:
  - Sugar: ≤ 25 g per serving (no added sugars)
  - Fat: ≤ 15 g per serving
- **Sodium**: ≤ 2400 mg per serving

The chosen validation metrics, such as calories, fiber, and sustainability, align with dietary guidelines for managing obesity and type 2 diabetes. These parameters ensure the recipes are nutritionally balanced while promoting environmentally conscious food choices. The virtual nutritionist ensures that generated recipes always meet these criteria. The validation model, in contrast, evaluates how the LLM performs without the virtual nutritionist’s iterative refinements, providing insights into its independent behavior. Sugar and fat thresholds were derived from Dutch dietary guidelines and tailored to individual user preferences. Added sugars and artificial sweeteners were excluded to prioritize natural sweetness from whole fruits. The thresholds are customizable to accommodate stricter requirements, such as enhanced carbohydrate control for diabetes management.

#### Sustainability validation.

Ingredients are evaluated for seasonality and local sourcing to minimize environmental impact. Seasonal ingredients, prioritized using the EUFIC database, receive higher sustainability ratings, while out-of-season or imported ingredients are flagged and rated lower.

#### Simulating real-world conditions.

To mimic real-world variability, a 20% randomized error rate was applied during the validation process. This accounts for inconsistencies such as ingredient availability and user input errors, ensuring the system remains adaptable to practical scenarios.

### Generation and validation of batch recipes

The system can generate and validate large batches of smoothie recipes based on random combinations of user inputs (age, BMI, dietary preferences). Each recipe is validated against health and sustainability criteria, with the following key information recorded:

- User Input: Age, BMI, and dietary preferences.
- Ingredients: Seasonal and sustainable choices.
- Nutritional Information: Calories, carbohydrates, fiber, sugars, fats, and sodium.
- Health and Eco-Rating: Based on nutritional and sustainability validation.
- Validation Status: Whether the recipe passes the validation criteria.

The results are stored in a CSV file containing details of 1,000 generated recipes, offering insights into the recipe generation and validation process.

### Metrics for model evaluation

The system’s performance was evaluated using two main metrics:

1. **Nutritional Validation Success Rate:** Achieved 80.1%. Failures were primarily due to high natural sugar content (e.g., bananas) and insufficient fiber levels.
2. **Sustainability Validation Success Rate:** Achieved 92%. Failures mainly resulted from using out-of-season or imported ingredients, impacting the recipes’ life cycle assessment (LCA).

These success rates and associated challenges are summarized in Table 1, providing an overview of the system’s ability to meet health and sustainability standards.

**Table 1:** Nutritional and sustainability validation results for generated smoothie recipes.

Category	Success Rate (%)	Key Challenges	Primary Cause of Invalidation
Nutritional Validation	80.1	High natural sugar content in some fruits (e.g., bananas), Low fiber content in some recipes	Excessive sugar or carbohydrate content
Sustainability Validation	92	Some recipes contained out-of-season or imported ingredients	Inclusion of out-of-season or non-local ingredients

### Real-time nutrients and seasonal considerations

The system uses a dynamic retrieval of nutrient data from the USDA FoodData Central API to ensure that the nutrient content of each ingredient is accurate and up-to-date. This real-time approach ensures that ingredient nutrient profiles match current data and that generated recipes meet Dutch dietary guidelines for type 2 diabetes and obesity. By using seasonal ingredients, the system also ensures that the recipes promote sustainability and minimize environmental impact.

### Ethics statement

This study does not involve human participants or live subjects. All data used in this research were obtained from publicly available, open-source datasets, and therefore, ethical approval was not required.

## Results and discussion

This study assesses the ability of the Retrieval-Augmented Generation (RAG) model to generate personalized smoothie recipes tailored for individuals with obesity and type 2 diabetes. The model successfully produced recipes that adhered to both nutritional and sustainability guidelines, demonstrating its potential as a valuable tool for dietary recommendations. This capability highlights its promise for addressing public health challenges by providing accessible, scalable solutions for managing chronic diseases like obesity and type 2 diabetes in diverse populations. To ensure suitability for the target audience, the recipes underwent a two-stage validation process focusing on compliance with nutritional standards and environmental sustainability.

### Performance of the RAG model

In creating 1,000 smoothie recipes, the model achieved an 80.1% success rate in nutrition validation and a 92% success rate in sustainability validation (Table 1). The 80.1% success rate highlights the system’s ability to generate balanced recipes. Failures due to high natural sugars and low fiber suggest incorporating more fiber-rich ingredients and stricter glycemic assessments to enhance effectiveness. Nutritional validation failures were mainly due to high natural sugars in fruits like bananas and mangoes, highlighting the need for stricter ingredient selection to support glycemic control.

The system did a good job of limiting added sugars and favoring healthy fats, e.g., from nuts and seeds, which was in line with dietary guidelines for people with obesity and type 2 diabetes. However, fiber content was a recurring challenge, with many recipes only slightly exceeded the minimum requirement of 8 grams. This could be remedied by including more high-fiber ingredients, such as chia seeds, flax seeds or leafy greens, in future versions of the recipe database.

The iterative validation process, powered by the virtual nutritionist, played a critical role in achieving these results by ensuring 100% compliance during recipe generation. Additionally, the validation model was used to assess the LLM’s independent performance, offering insights into its recipe generation behavior without the virtual nutritionist’s adjustments. This dual approach highlights the robustness of the system. This approach contrasts with static validation methods, where recipes are evaluated only once, and demonstrates the value of integrating adaptive validation for real-world applications.

### Nutritional analysis

The model successfully generated recipes that met the thresholds for calories, carbohydrates, sugar, and fat, demonstrating its ability to provide nutritionally sound options. Table 2 highlights the validation process, showcasing key parameters evaluated for compliance with dietary guidelines. Fiber content, critical for glycemic control and satiety in type 2 diabetes, was emphasized separately. However, both valid and invalid recipes showed limitations in fiber inclusion, suggesting the need for refining the recipe database to prioritize high-fiber ingredients.

**Table 2:** Comparison of validated smoothie recipes against dietary thresholds, highlighting key parameters such as calories, carbohydrates, fiber, sugar, fat, and sodium.

Nutrient	Per Serving	Validation Threshold	Status
Calories (kcal)	350	≤ 350	Valid
Carbohydrates (g)	50	≤ 50	Valid
Sugar (g)	25	≤ 25	Valid
Fiber (g)	8	≥ 8	Just Above Minimum
Fat (g)	15	≤ 15	Valid
Sodium (mg)	2400	≤ 2400	Valid

Invalid recipes were primarily disqualified due to excessive sugar content, reflecting challenges posed by naturally occurring sugars in common ingredients. Most violations exceeded limits by small margins (10–20%), but a minority (approximately 5%) significantly exceeded thresholds, such as surpassing sugar limits by over 50%. These cases highlight the need for stricter ingredient selection and validation mechanisms, particularly for high-risk parameters.

While smoothies are a convenient way to deliver personalized nutrition, they may cause higher blood glucose spikes than whole fruits due to faster absorption. To address this, the system prioritizes low-glycemic ingredients and balanced macronutrient compositions, incorporating fiber-rich and protein-based components to slow digestion and reduce glycemic impact. Future improvements should focus on enhancing fiber content and sugar control.

The system achieved an 80.1% adherence rate to nutritional guidelines—a promising outcome for a prototype tackling complex dietary requirements. However, further refinements are needed to improve this performance. Enhancing ingredient selection, incorporating advanced glycemic load analysis, and exploring alternative LLM setups could help achieve closer to 90% adherence, improving the system’s reliability and suitability for real-world use. Compared to prior models such as MOPI-HFRS and NGQA, our approach extends beyond personalization by embedding sustainability constraints and an iterative refinement mechanism. The virtual nutritionist ensures clinical compliance during generation, while the RAG framework grounds responses in validated nutritional data. This combination enables not just personalization, but explainable and reliable recommendations that can be translated into clinical and telehealth workflows.

### Influence of dietary preferences on the validity of recipes

**Fig 3:**
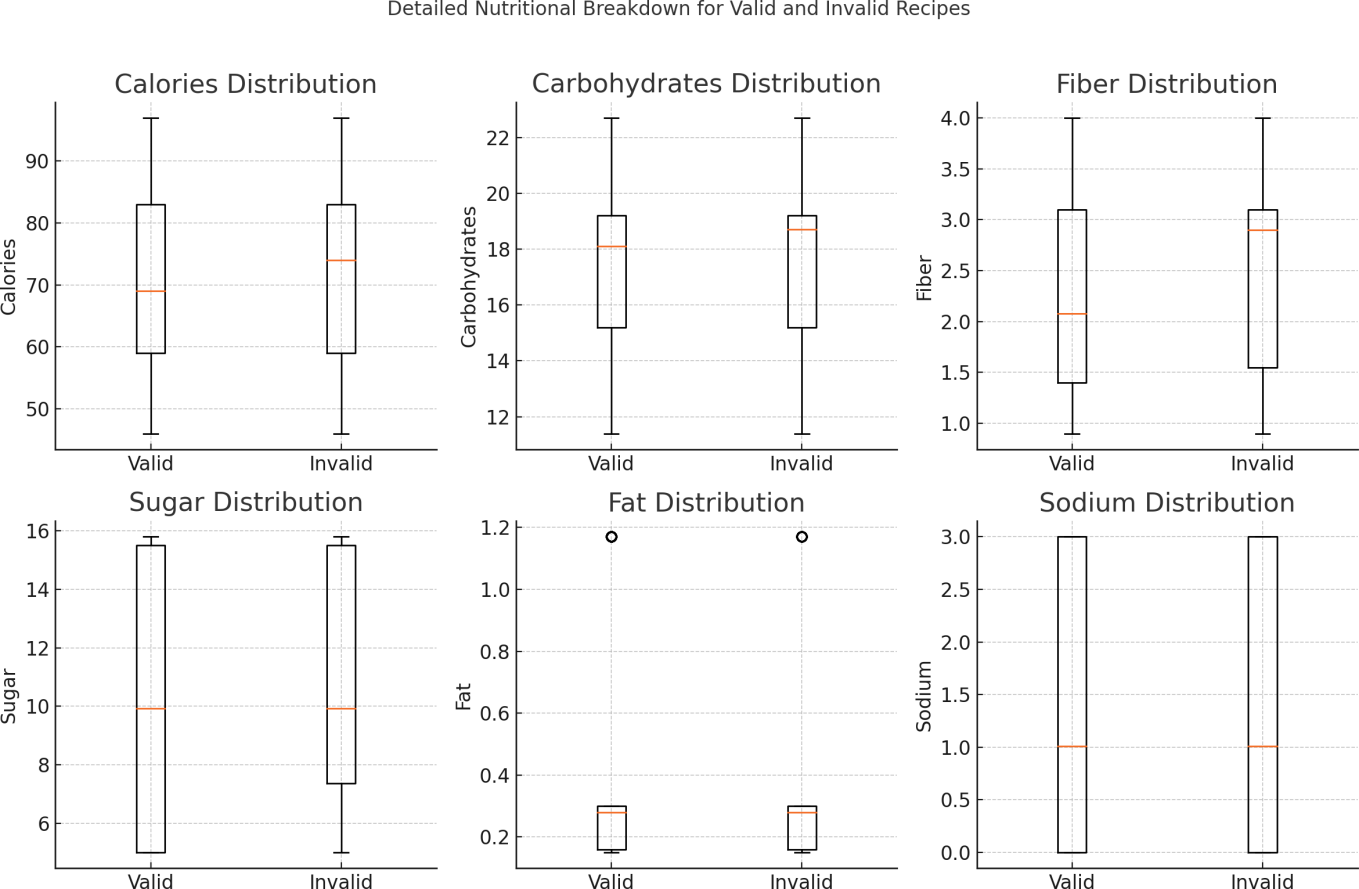
Nutritional distribution comparisons between valid and invalid smoothie recipes.

The Fig 3 compares the nutritional distribution of valid and invalid smoothie recipes across calories, carbohydrates, fiber, sugar, fat, and sodium. Overall, valid and invalid recipes show similar distributions in calories, carbohydrates, fiber, and sodium. However, sugar levels exhibit more variability in invalid recipes, and fat content shows slight deviations with outliers in invalid ones, indicating potential inconsistencies in these areas. Fruits are generally better fiber contributor compared to vegetables [18].

### Sustainability analysis

The sustainability test was very successful: 92% of valid recipes contained seasonal, locally available ingredients. Recipes that contained out-of-season ingredients, such as tropical fruits in winter, were marked with a note that they should be changed or replaced. By prioritizing seasonal ingredients, the system not only reduces the carbon footprint associated with food production but also encourages users to adopt environmentally sustainable eating habits. This focus aligns dietary recommendations with broader climate action goals. This focus on sustainability supports the environmental goals of the study by encouraging the use of local and seasonal produce to reduce the carbon footprint of food production (Table 3).

**Table 3:** Average nutritional comparison of valid and invalid smoothie recipes against dietary thresholds.

Nutrient	Valid Recipe (Avg.)	Invalid Recipe (Avg.)	Threshold
Calories (kcal)	340	380	≤ 350
Carbohydrates (g)	48	60	≤ 50
Sugar (g)	22	30	≤ 25
Fiber (g)	9	7	≥ 8
Fat (g)	13	17	≤ 15
Sodium (mg)	2200	2600	≤ 2400

In addition, in most recipes, dairy-based drinks were replaced with eco-friendly alternatives such as tap water and almond milk [19]. This switch to plant-based beverages is in line with the principles of sustainability and helps to minimize the environmental impact of the recipes produced [20]. By promoting the use of whole fruits and vegetables, the system has the potential to reduce food waste, though this remains to be validated in future studies.

### Limitations and possibilities for improvement

Despite its success in generating nutritionally balanced and environmentally sustainable recipes, the system has several limitations that need to be addressed:

#### Fiber content.

While most recipes met the minimum threshold of 8 grams of fiber, many only slightly exceeded this value. Future iterations should prioritize high-fiber ingredients, such as seeds and leafy greens, to better support the nutritional needs of individuals with obesity and diabetes.

#### Dealing with naturally occurring sugars.

Although added sugars were well controlled, the system struggled to manage naturally occurring sugars in fruits like bananas and mangoes. Incorporating advanced techniques, such as glycemic load analysis, could improve recipe suitability for individuals with diabetes.

#### Sustainable substitutions.

The system effectively flagged recipes containing out-of-season ingredients but lacked the ability to automatically suggest sustainable alternatives. Adding a substitution mechanism to replace unsustainable ingredients could enhance the system’s overall sustainability.

#### Randomized error mechanism.

The 20% randomized error mechanism introduced variability to simulate real-world inconsistencies, such as ingredient availability or user input errors. While this added realism, further research is needed to determine whether it improves system reliability or unnecessarily complicates the validation process.

The virtual nutritionist’s reliance on computational resources may limit scalability in high-demand settings. Optimizing validation efficiency and exploring distributed computing can address these challenges while ensuring data privacy. Additionally, studies on long-term adherence to AI-driven dietary recommendations, integration with electronic health records (EHRs), and real-world validation in diverse clinical settings are critical to enhancing the system’s impact and scalability.

### Implications and future work

This study demonstrates the successful implementation of a Retrieval-Augmented Generation (RAG) system that translates Dutch dietary guidelines into actionable smoothie recipes for managing obesity and type 2 diabetes. The validation framework effectively converts complex nutritional guidelines into quantifiable recipe parameters, providing a foundation for integration into real-world healthcare settings, such as telemedicine platforms or chronic disease management programs. This adaptability enhances its potential to improve patient adherence and support clinician-led dietary interventions.

While valid recipes consistently maintain carbohydrates (48g), sugars (22g), and fiber (9g) within therapeutic ranges, invalid recipes highlight challenges in controlling carbohydrates, sugars, and fats. These findings point to areas for improvement, such as ingredient selection and portion optimization. Notably, the system balances multiple nutritional requirements while maintaining recipe practicality, demonstrating its potential for managing chronic diseases.

The system dynamically integrates diverse datasets, including seasonal ingredients and sustainability guidelines, offering greater flexibility compared to static, rule-based models. While the current implementation is tailored to Dutch dietary guidelines, the framework can be adapted to other populations by integrating region-specific dietary recommendations and sustainability data. Future iterations should focus on expanding the ingredient database to include culturally diverse and region-specific foods, enhancing the system’s inclusivity and global applicability. Dataset biases, such as overrepresentation of Western ingredients, may limit recipe diversity, but expanding the dataset could improve inclusivity.

For practical implementation, the RAG-based system can be integrated into digital health platforms or telemedicine services to provide personalized nutritional guidance. This system has the potential to not only support individual dietary management but also inform public health policies by promoting sustainable dietary practices. Future research could explore integration with population-level health monitoring systems and its role in reducing healthcare disparities. However, real-world deployment requires addressing privacy concerns, adhering to regulations like GDPR, and obtaining certifications such as CE marking or FDA approval. Transparent AI decision-making and continuous validation with real-world data will be essential for building trust and refining system performance.

The system is designed with scalability in mind, using efficient embedding techniques and local deployment of the LLaMA3 model to minimize latency. Future iterations may include distributed computing or cloud-based solutions to handle increased user demand while maintaining privacy standards.

Future development should focus on expanding the ingredient database, enhancing sustainability validation, and integrating user feedback mechanisms. These improvements will strengthen the system’s practical utility in clinical settings. Validation studies with healthcare providers and patients will be essential to assess usability, dietary adherence, and clinical outcomes, refining the system for real-world adoption.

**Fig 4:**
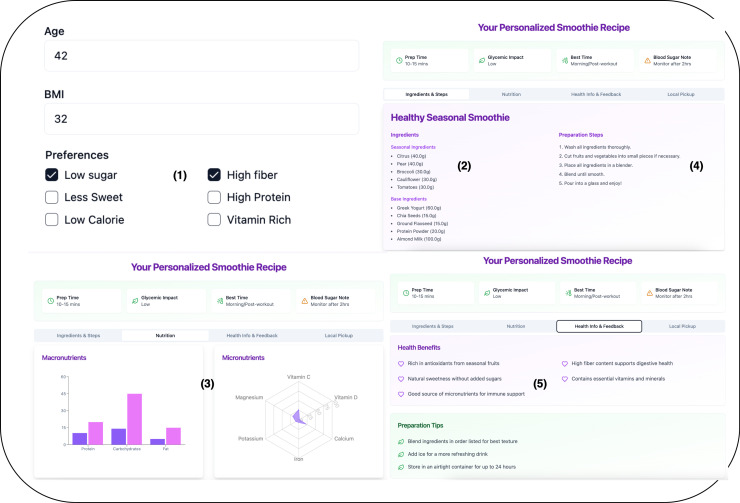
User interface of the personalized smoothie recipe generator.

A user-friendly web application (Fig 4) was also developed, enabling users to input their age, BMI, and dietary preferences for real-time customized recommendations. As shown in Fig 4, the interface consists of: (1) Input form for age, BMI, and dietary preferences, (2) Personalized smoothie recipe output with seasonal ingredient list (3) Nutritional breakdown showing calories, macronutrients, and fiber (4) Preparation Instruction (5) Explanation of health benefits and ingredient rationale.

Future enhancements could include real-time health data from wearable devices and additional personalization parameters, such as activity levels and metabolic biomarkers, to provide even more tailored dietary recommendations. These advancements could improve the system’s clinical utility and support better health outcomes. The system’s scalability and privacy features make it adaptable for diverse populations and telemedicine platforms, supporting broader chronic disease management.

## Conclusion

This study demonstrates the potential of AI-driven dietary recommendation systems to transform chronic disease management by enhancing patient self-management, improving adherence to dietary guidelines, and reducing the burden on healthcare systems through scalable and evidence-based interventions. Our Retrieval-Augmented Generation (RAG) model provides personalized, nutritionally balanced, and sustainable smoothie recipes, benefiting healthcare providers and patients alike. By adhering to nutritional guidelines and incorporating eco-friendly ingredients, the model empowers users to make informed dietary choices that align with health and sustainability goals.

A key strength of this system lies in enhancing health literacy by transforming complex nutritional guidelines into accessible, actionable insights, empowering users to make informed dietary choices. The user-friendly web application enhances this accessibility, offering real-time, customized dietary guidance through a straightforward interface.

Aligned with Sustainable Development Goals (SDGs) and circular economy principles, this approach demonstrates the value of integrating health and sustainability in digital health solutions. Future development will focus on enhancing scalability and telemedicine integration, enabling broader adoption in health management. Incorporating real-time health data and advanced personalization will further improve accessibility and outcomes for diverse populations. The system could be implemented in community health programs, providing accessible dietary recommendations in underserved areas. Additionally, its adaptability to clinical workflows positions it as a valuable tool for dietitians managing patients with chronic conditions.

## Supporting information

S1 Text Sustainable dietary guidelines. This file provides the environmental sustainability guidelines for the Dutch diet, sourced from the National Institute for Public Health and the Environment (RIVM). It emphasizes plant-based, seasonal, and low-impact food choices, integrated into the smoothie recipe generation system to ensure recipes align with health and sustainability goals, supporting the United Nations Sustainable Development Goals (SDGs).
(DOCX)

S2 Text Dutch dietary guidelines for type 2 diabetes. This file contains the Dutch dietary guidelines for managing type 2 diabetes and obesity, sourced from the Health Council of the Netherlands. It details recommendations on macronutrient distribution, glycemic index control, fiber intake, and specific food groups (e.g., fruits, vegetables, whole grains), used to ensure nutritional accuracy in personalized smoothie recipes.
(DOCX)

S3 Text Curated smoothie recipes. This file includes a curated collection of diabetes-friendly smoothie recipes from reputable sources, cross-referenced with the USDA FoodData Central database. These recipes serve as a reference for nutritional data and validation, supporting the system’s ability to generate health-compliant smoothie recipes for individuals with obesity and type 2 diabetes.
(DOCX)

S4 Text Prompt examples for recipe generation. This file presents the system and user prompt templates used in the Retrieval-Augmented Generation (RAG) system. It illustrates how user inputs (age, BMI, dietary preferences) and contextual data from nutritional and sustainability guidelines are processed to generate personalized, evidence-based smoothie recipes.
(DOCX)
