# Supplementary material for: AI-driven personalized nutrition: RAG-based digital health solution for obesity and type 2 diabetes
Source: PLOS Digit Health. 2025 May 6;4(5):e0000758. doi: 10.1371/journal.pdig.0000758 (PMC12054865; doi:10.1371/journal.pdig.0000758)
Supplement: S1 Text — This file provides the environmental sustainability guidelines for the Dutch diet, sourced from the National Institute for Public Health and the Environment (RIVM). It emphasizes plant-based, seasonal, and low-impact food choices, integrated into the smoothie recipe generation system to ensure recipes align with health and sustainability goals, supporting the United Nations Sustainable Development Goals (SDGs). (DOCX) [file pdig.0000758.s001.docx]

**S1_Text: Safe Healthy and Sustainable Diets in the Netherlands**

- [(*Advisory Report on Dutch Dietary Guidelines for type 2 diabetics*, n.d.)](https://www.rivm.nl/bibliotheek/rapporten/2016-0198.pdf)
- <https://www.rivm.nl/publicaties/environmental-sustainability-of-dutch-diet-background-report-to-what-is-on-our-plate>
