# Supplementary material for: AI-driven personalized nutrition: RAG-based digital health solution for obesity and type 2 diabetes
Source: PLOS Digit Health. 2025 May 6;4(5):e0000758. doi: 10.1371/journal.pdig.0000758 (PMC12054865; doi:10.1371/journal.pdig.0000758)
Supplement: S2 Text — This file contains the Dutch dietary guidelines for managing type 2 diabetes and obesity, sourced from the Health Council of the Netherlands. It details recommendations on macronutrient distribution, glycemic index control, fiber intake, and specific food groups (e.g., fruits, vegetables, whole grains), used to ensure nutritional accuracy in personalized smoothie recipes. (DOCX) [file pdig.0000758.s002.docx]

**S2 Text: Dutch Dietary Guidelines for Obesity and Type 2 Diabetics**

- <https://www.healthcouncil.nl/binaries/healthcouncil/documenten/advisory-reports/2021/11/16/dutch-dietary-guidelines-for-people-with-type-2-diabetes/Summary-Dutch-dietary-guidelines-for-people-with-type-2-diabetes.pdf>
- <https://www.healthcouncil.nl/binaries/healthcouncil/documenten/advisory-reports/2021/11/16/dutch-dietary-guidelines-for-people-with-type-2-diabetes/Advisory-report-Dutch-dietary-guidelines-for-people-with-type-2-diabetes.pdf>
- <https://www.healthcouncil.nl/binaries/healthcouncil/documenten/advisory-reports/2021/11/16/dutch-dietary-guidelines-for-people-with-type-2-diabetes/DDG-Diabetes-type2-A_Methodology-for-the-evaluation-of-evidence.pdf>
- <https://www.healthcouncil.nl/binaries/healthcouncil/documenten/advisory-reports/2021/11/16/dutch-dietary-guidelines-for-people-with-type-2-diabetes/DDG-Diabetes-type2-B_Fruit-and-vegetables.pdf>
- <https://www.healthcouncil.nl/binaries/healthcouncil/documenten/advisory-reports/2021/11/16/dutch-dietary-guidelines-for-people-with-type-2-diabetes/DDG-Diabetes-type2-C_Whole-grain-foods.pdf>
- <https://www.healthcouncil.nl/binaries/healthcouncil/documenten/advisory-reports/2021/11/16/dutch-dietary-guidelines-for-people-with-type-2-diabetes/DDG-Diabetes-type2-D_Dietary-fibre.pdf>
- <https://www.healthcouncil.nl/binaries/healthcouncil/documenten/advisory-reports/2021/11/16/dutch-dietary-guidelines-for-people-with-type-2-diabetes/DDG-Diabetes-type2-E_Legumes.pdf>
- <https://www.healthcouncil.nl/binaries/healthcouncil/documenten/advisory-reports/2021/11/16/dutch-dietary-guidelines-for-people-with-type-2-diabetes/DDG-Diabetes-type2-F_Beverages-with-added-sugar.pdf>
- <https://www.healthcouncil.nl/binaries/healthcouncil/documenten/advisory-reports/2021/11/16/dutch-dietary-guidelines-for-people-with-type-2-diabetes/DDG-Diabetes-type2-G_Dairy-products.pdf>
- <https://www.healthcouncil.nl/binaries/healthcouncil/documenten/advisory-reports/2021/11/16/dutch-dietary-guidelines-for-people-with-type-2-diabetes/DDG-Diabetes-type2-H_Coffee.pdf>
- <https://www.healthcouncil.nl/binaries/healthcouncil/documenten/advisory-reports/2021/11/16/dutch-dietary-guidelines-for-people-with-type-2-diabetes/DDG-Diabetes-type2-I_Sodium.pdf>
- <https://www.healthcouncil.nl/binaries/healthcouncil/documenten/advisory-reports/2021/11/16/dutch-dietary-guidelines-for-people-with-type-2-diabetes/DDG-Diabetes-type2-J_Carbohydrate+and+fat+substitutions.pdf>
- https://www.healthcouncil.nl/binaries/healthcouncil/documenten/advisory-reports/2021/11/16/dutch-dietary-guidelines-for-people-with-type-2-diabetes/DDG-Diabetes-type2-K_Reduced+carbohydrate+diets.pdf
