# Supplementary material for: AI-driven personalized nutrition: RAG-based digital health solution for obesity and type 2 diabetes
Source: PLOS Digit Health. 2025 May 6;4(5):e0000758. doi: 10.1371/journal.pdig.0000758 (PMC12054865; doi:10.1371/journal.pdig.0000758)
Supplement: S3 Text — This file includes a curated collection of diabetes-friendly smoothie recipes from reputable sources, cross-referenced with the USDA FoodData Central database. These recipes serve as a reference for nutritional data and validation, supporting the system’s ability to generate health-compliant smoothie recipes for individuals with obesity and type 2 diabetes. (DOCX) [file pdig.0000758.s003.docx]

**S3 Text: Curated Smoothie Recipes for Obesity and Type 2 Diabetics**

- https://diabetesfoodhub.org/recipes/pumpkin-pie-smoothie
- https://diabetesfoodhub.org/recipes/chocolate-cherry-smoothie
- https://diabetesfoodhub.org/recipes/peach-cream-smoothie
- https://diabetesfoodhub.org/recipes/kid-friendly-raspberry-smoothie-pops
- https://diabetesfoodhub.org/recipes/smoothie-breakfast-bowl
- https://diabetesfoodhub.org/recipes/blueberry-green-tea-smoothie
- https://diabetesfoodhub.org/recipes/tropical-smoothie-bowl
- https://diabetesfoodhub.org/recipes/green-smoothie-bowl
- https://diabetesfoodhub.org/recipes/fruit-and-almond-smoothie
- https://diabetesfoodhub.org/recipes/roasted-indian-cauliflower-tossed-chickpeas-and-cashews
- https://diabetesfoodhub.org/recipes/milk-chocolate-peanut-butter-banana-smoothie
- https://diabetesfoodhub.org/recipes/summer-fruit-smoothie
- https://diabetesfoodhub.org/recipes/superfood-smoothie
- https://diabetesfoodhub.org/recipes/almond-wild-blueberry-and-flax-smoothie
- https://diabetesfoodhub.org/recipes/moroccan-avocado-smoothie
- https://www.healthline.com/health/diabetes/diabetic-friendly-smoothies#berry-nut-milkshake
- https://ninacheriefranklin.com/healthy-living/low-sugar-diabetes-friendly-smoothie-recipes/
- https://www.healthshots.com/healthy-eating/recipes/diabetes-friendly-smoothies/
- https://www.medicalnewstoday.com/articles/317388#healthful-fats
- https://www.freedomfromdiabetes.org/blog/post/start-your-morning-with-green-smoothie-&-control-your-diabetes/1405
- https://erinpalinski.com/diabetic-smoothie-recipes
