# Supplementary material for: AI-driven personalized nutrition: RAG-based digital health solution for obesity and type 2 diabetes
Source: PLOS Digit Health. 2025 May 6;4(5):e0000758. doi: 10.1371/journal.pdig.0000758 (PMC12054865; doi:10.1371/journal.pdig.0000758)
Supplement: S4 Text — This file presents the system and user prompt templates used in the Retrieval-Augmented Generation (RAG) system. It illustrates how user inputs (age, BMI, dietary preferences) and contextual data from nutritional and sustainability guidelines are processed to generate personalized, evidence-based smoothie recipes. (DOCX) [file pdig.0000758.s004.docx]

**S4 Text: System Prompt and User Prompt – Dynamically constructed**

"""

Generate a personalized smoothie recipe for an individual with obesity and type 2 diabetes. The recipe should be tailored based on the following inputs:

Age: {age} years old.

BMI: {bmi}.

Dietary Preferences: {preferences}.

Using the following context and data from Open Food Facts, Sustainable Dutch Diet and RIVM Guidelines of diet for people with Obesity and type 2 diabetics, create a smoothie recipe that is:

- Nutritionally balanced to support weight management and blood sugar control.

- Low in glycemic index and high in essential nutrients.

Context: {context}

Include the following details:

- Ingredients: List each ingredient with its quantity.

- Nutritional Breakdown: Provide the smoothie's nutritional information, including calories, carbohydrates, fiber, protein, fat, and sugar content.

- Preparation Instructions: Step-by-step instructions for making the smoothie.

- Explainable Component: A brief explanation of how the chosen ingredients benefit the individual based on their age, BMI, and dietary preferences. Explain how the smoothie supports weight management and blood sugar control, referencing relevant data or research from the sources used.

"""
